# Development and Validation of an Online Survey to Assess Perception of Diabetes Risk and Barriers and Facilitators to Weight Loss Following Gestational Diabetes

**DOI:** 10.3390/ijerph18020480

**Published:** 2021-01-08

**Authors:** Kristy L. Gray, Mayya Grebenshchikova, Sharleen L. O’Reilly, Lois McKellar, Peter M. Clifton, Jennifer B. Keogh

**Affiliations:** 1 Clinical and Health Sciences, University of South Australia, Adelaide, SA 5000, Australia; gremy011@mymail.unisa.edu.au (M.G.); Lois.McKellar@unisa.edu.au (L.M.); Peter.Clifton@unisa.edu.au (P.M.C.); Jennifer.Keogh@unisa.edu.au (J.B.K.); 2 School of Agriculture and Food Science, University College Dublin, Belfield, D04 V1W8 Dublin, Ireland; sharleen.oreilly@ucd.ie

**Keywords:** survey development, gestational diabetes, diabetes prevention, content validation, Theoretical Domains Framework

## Abstract

Our objective was to describe the development and validation of a survey investigating barriers to weight loss, perception of diabetes risk, and views of diet strategies following gestational diabetes (GDM). The survey underwent three stages of development: generation of items, expert evaluation, and pilot testing. A content validation index (CVI) was calculated from expert responses regarding item relevance, coherence, clarity, and response options. Experts also responded to the domain fit of questions linked to the Theoretical Domains Framework (TDF). Pilot responders answered the survey and responded to review questions. Six experts in the field of nutrition, midwifery, psychology, or other health or medical research completed the expert review stage of the survey. In the pilot test, there were 20 responders who were women with previous GDM and who were living in Australia. The overall CVI from the expert review was 0.91. All questions except one received an I-CVI of >0.78 for relevance (*n* = 35). Fourteen of the 27 items linked to the TDF received an agreement ratio of <1.0. Twenty-seven of the 31 pilot questions were completed by ≥90% of responders. Pilot review questions revealed an agreement percentage of ≥86% (*n* = 12) regarding the survey’s ease to complete, understand, importance, length, and interest level. The final survey tool consists of 30 items and achieved content validation through expert evaluation and pilot testing.

## 1. Introduction

Diabetes mellitus poses a major public health problem around the world, affecting approximately 463 million people and accounting for 4.2 million deaths in 2019 [1]. Type 2 diabetes (T2DM) accounts for around 90% of all diabetes cases [1]. Gestational diabetes (GDM) affected 16% of pregnancies resulting in live births in the world in 2019 [1] and is associated with a risk of developing T2DM in the years following pregnancy, which is 10 times higher than women who do not develop GDM in pregnancy [2]. Women who have GDM and are in an overweight or obese category are twice as likely to develop T2DM than women with previous GDM who are in a healthy weight range [3,4]; therefore, weight loss after GDM is a vital part of diabetes prevention. Women who develop GDM that are in a healthy BMI range should aim to return to their pre-pregnancy weight following pregnancy and maintain a healthy lifestyle to minimize risk of T2DM development [5]. Previous research has investigated barriers to weight loss and the perception of T2DM risk following GDM; however, these studies typically target women in the first one to two years following birth [6,7,8]. Much of the research around lifestyle behaviors and barriers to weight loss after GDM has come from semi-structured interviews and focus groups [8]. There are limited large studies that investigate the risk perception of T2DM and barriers to weight loss beyond this immediate postpartum period. There is also limited research investigating the weight loss strategies that women with previous GDM have tried and where they source information on weight loss following a GDM pregnancy. Theory-based health investigations allow researchers to identify which determinants of behavior are most relevant in the target population and design interventions that will appropriately address barriers and increase the likelihood of successful behavior change [9]. The Theoretical Domains Framework (TDF) is a theoretical framework consisting of 14 domains to classify attitudes and behaviors, barriers, and facilitators to behavior change [10]. This survey sought to explore women’s perception of their T2DM risk after GDM and use the TDF to identify barriers to weight loss and identify diet strategies, programs, or services that these women feel would be suitable at any time after GDM. Opinions of an intermittent ‘5:2’ diet were of interest. This paper presents the survey development and review process used to achieve content validation prior to the final survey administration. The final survey was designed to be an exploratory, cross-sectional online survey of women living in Australia with previous GDM.

## 2. Materials and Methods

There were three stages to the development and content validation of the survey: initial survey development, expert evaluation, and pilot study testing.

Stage 1: Survey Development

The survey was developed by an accredited dietitian in October 2017 following a review of literature on diabetes prevention after GDM, which revealed limited large studies past 12 months post-pregnancy that investigated barriers to weight loss, diet strategies or services, or perception of diabetes risk following GDM. The TDF was used to guide the development of the survey and categorize beliefs, barriers to weight loss, and perception of diabetes risk as well as structure for the data analysis plan. Table A1 (Appendix A) shows the TDF domains. The survey was developed using the LimeSurvey software package (LimeSurvey, Hamburg, Germany) and contained 36 questions including yes/no multiple-choice and Likert-scale rankings with optional open text comments for several questions. There were five parts to the survey: (1) demographic information and personal characteristics, (2) perception of diabetes risk and views on weight loss after pregnancy; (3) barriers to weight loss; (4) opinions of diet strategies and services; and (5) opinions and use of an intermittent two-day diet (the ‘5:2’ diet). Opinions of an intermittent diet were of interest, as this study was part of a larger research project investigating intermittent energy restriction for weight loss in overweight women with previous gestational diabetes. A plain-language approach was used for questions, and the level of readability was assessed using the Gobbledygook formula, determined to be at a Grade 10 reading level (15–16 years old) [11]. Six questions were linked to the TDF. Questions regarding barriers to weight loss, perception of diabetes risk, and opinions of diet strategies were more heavily informed by the TDF. Question 29 asked participants to respond with their level of agreement to a list of 22 barriers, facilitators, and opinions regarding weight loss. The statements were mapped to a specific TDF domain by the primary researcher.

Stage 2: Expert Review

Invitations to participate in the survey expert review stage were sent via email to 50 recognized experts in the fields of nutrition, midwifery, psychology, or other health or medical research, with six experts completing the expert review (12% response rate). A sample of 3–10 expert reviewers is deemed adequate for content validation of a new survey [12]. Experts were identified from the author lists of journal articles in relevant areas and searches on various university departmental staff pages. Of the six reviewers, four were Accredited Practicing Dietitians, one had a background in midwifery and nursing, and one a background in health and exercise psychology and behavior. All six reviewers worked in research and academia settings. Recruitment occurred between November and December 2017. An email invitation outlining the study with a link to the online survey, instructions for rating each item, and an information sheet outlining the TDF domains was sent to each potential reviewer. Reviewers were asked to respond to the relevance, clarity, coherence, and answer options for each question on a four-point Likert scale [13]. The definition of each item and scoring was provided to reviewers as follows: relevance (Is the question going to help achieve an answer to the research objectives?), clarity (Is the question clear and specific? Does it make sense to the reader?), coherence (Is this question logical, consistent, and reasonable in the context of the research problem being addressed?), and answer options (Are the answers appropriate for the question being asked?). The answer options for the four-point Likert scale were 1 = not acceptable, 2 = below expectations, 3 = meets expectations, and 4 = exceeds expectations. Reviewers were also asked “Is there anything else you would like to say about the question (e.g., item rewording, answer order/options, or anything else).” For the TDF-linked questions, reviewers were also asked to rate the fit of the identified TDF domain on a four-point Likert scale (1 = absolutely does not fit, 2 = doubtful fit, 3 = fair fit, 4 = exact fit). At the end of the survey, reviewers were asked for any other overall comments or feedback about the survey.

Stage 3: Pilot Test

Following changes made to the survey from the expert review, a pilot test was undertaken to evaluate the usability of the survey, identify any procedural problems, and receive overall survey feedback from participants. The inclusion criteria for the pilot and final study were the same: women with previous GDM, living in Australia, aged 18 years or older, and no diagnosis of diabetes prior to GDM. Recruitment occurred between December 2017 and January 2018 and was achieved through a social media advertisement. A convenience sample of 21 women participated in the pilot survey, but *n* = 1 was ineligible. Recruitment was through social media advertisement until the required number of responses were received (*n* = 20), and as such, a response rate could not be determined. A sample size of *n* = 20 was deemed adequate based on recommendations by Julius (2005), who specified a minimum of 12 participants per group needed to pilot a study [14]. Comparable numbers have been used to pilot cross-sectional surveys in similar populations [15,16,17]. Participants responded to the survey questions and an additional question covering how easy the survey was to understand and complete as well as the survey’s length, importance, and if it was interesting using a 5-point agreement Likert scale. Participants were also asked to provide any extra comments or feedback as free text.

### 2.1. Statistical Analysis

#### 2.1.1. Expert Review

Results from the expert review stage were used to determine the initial retention, deletion, and modification of survey items. The content validation index (CVI) is an inter-rater agreement calculated based on expert reviewers’ ratings [12]. It is a widely used tool for quantifying the relevance of a multi-item tool to the construct of interest and commonly used in the development of questionnaires and surveys in health and nursing research [12]. The content validation of each item was performed by a panel of experts (*n* = 6) and was achieved through calculating the content validation index (I-CVI) (number of expert answers with a score of 3 or 4 divided by the total number of expert answers) in the dimensions of relevance, clarity, coherence, and answer options [12]. An overall CVI of the survey was also determined by calculating the mean index from the I-CVI relevance index. A CVI of at least 0.83 is commonly considered acceptable when six expert reviewers rate the items, reflecting one expert who disagreed and five who agreed the question was appropriate in the dimension being measured. A CVI of ≤0.78 reflects two experts disagreeing and four agreeing, is considered unacceptable when there are six reviewers, and requires modification [12].

Data were analyzed using Microsoft Excel (Office 2016 for Microsoft, Redmond, WA, USA). Questions that received an I-CVI of ≤0.78 were modified or removed [12]. The TDF domain fit responses were analyzed by calculating the number of experts who scored the chosen domain fit at 3 (fair fit) or 4 (exact fit) divided by the number of expert reviewers. All open text comments for each question and the overall survey from the reviewers were read and changes were made based on the suggestions. Analysis of the expert review stage was performed using Microsoft Excel (Office 2016 for Microsoft, Redmond, WA, USA).

#### 2.1.2. Pilot Test

The pilot test determined the final retention, deletion, and modification of survey items. Data analysis was undertaken using IBM SPSS Statistical software v25 for Windows (IBM, Chicago, IL, USA) and Microsoft Excel (Office 2016 for Microsoft, Redmond, WA, USA). A descriptive analysis of the demographic information for the pilot test was also performed. Data were tested for normality using Q–Q plots, histograms, and Shapiro–Wilk tests. As most data were skewed data, they are presented as the median and IQR, unless otherwise stated. An answer percentage was calculated for each question (number of the responders who answered each question divided by the number of responders who were presented with the question, multiplied by 100). The percentage of participants who responded with a score of ≥4 (agree or strongly agree) for the positively phrased review questions or ≤2 (disagree or strongly disagree) for the negatively phrased review question was also calculated for each of the pilot test review questions.

#### 2.1.3. Ethics

The research was conducted according to the NHMRC National Statement on Ethical Conduct in Human Research. Ethics approval was granted by The University of South Australia’s Human Research Ethics Committee prior to the project commencing (Protocol No. 200231). Reviewers and pilot responders provided informed consent electronically prior to commencing the survey.

## 3. Results

### 3.1. Expert Review

The overall CVI was 0.91. For relevance, clarity, coherence, and response options, there were 1, 10, 3, and 12 items, respectively, that received an I-CVI score of ≤0.78. Table 1 shows the index scores for each survey item and the changes made. The one question that received an I-CVI score of ≤0.78 for relevance was a screening question needed to determine eligibility for the survey. The four eligibility screening questions were regrouped into one question at the start of the survey, which asked participants if they met all the eligibility requirements or not.

All 36 items received expert comments. As a result, 32 items were modified, and 10 items were merged into 4 items. The answer mode or question wording of four items that received acceptable I-CVIs across all measures were minimally modified based on expert-panel-written suggestions. Question order was also modified to improve survey flow, and the online platform was changed to Survey Monkey (SurveyMonkey Inc., San Mateo, CA, USA) to improve the user interface and allow for more specialized functions such as skip logic.

Fourteen of the 27 TDF items had an agreement ratio of <1.0 for the TDF framework fit review. The question that listed barriers to weight loss and asked responders to rate their agreement or disagreement with the statements was found to be too lengthy by reviewers, and some statements were repetitive; therefore, the question was reduced from 22 to 15 statements. Four items that were originally linked to the TDF domain ‘Environmental Context and Resources’ were changed to the domain ‘Beliefs about Capabilities’, one item that was linked to the ‘Behavioral Regulation’ domain was also changed to ‘Beliefs about Capabilities’, and two items that were linked to the domain ‘Knowledge’, were changed to the domains ‘Beliefs about Consequences’ and ‘Social/Professional Role and Identity’. Four of the barriers to weight loss statements that were negatively phrased were changed to be positively phrased based on reviewer suggestions that responders whose first language is not English may find these statements difficult to understand.

### 3.2. Pilot Test

Participants had a median (IQR) age of 36 (5) years (*n* = 20) and were 2 (4) years post-GDM (*n* = 19), with a BMI of 27.3 kg/m^2^ (11.7 kg/m^2^) (*n* = 18). Eleven and six women, respectively, were in the overweight or obese (61%) and healthy weight range (28%), and one woman was underweight (9%). Two participants did not provide their weight; therefore, their BMI could not be established.

**Figure 1:**
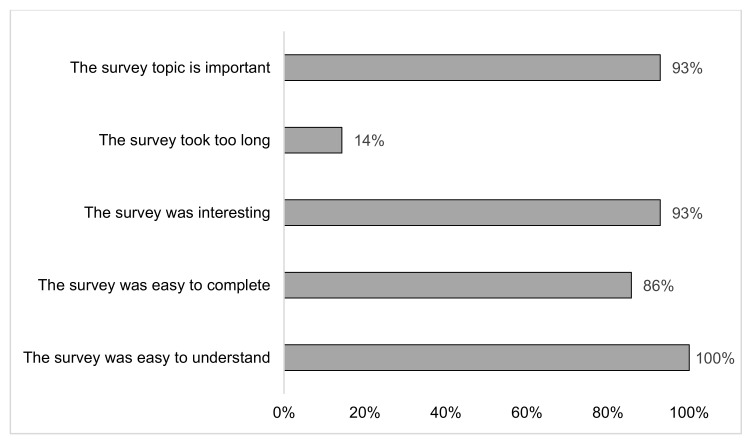
Pilot test review. Percentage of responders who agreed or strongly agreed on the Likert-scale survey review questions (*n* = 14); responses were received on a five-point Likert-scale (1 = strongly disagree, 2 = disagree, 3 = not sure, 4 = agree, 5 = strongly agree).

Twenty-seven questions were answered by ≥ 90% of responders. Skip logic was used for questions on diet types and intermittent dieting (Questions 23 and 28), and as a result, only 13 participants were shown the more detailed questions on diet types (Question 24), 15 on intermittent dieting (Question 29a), and five on the suitability of intermittent dieting (Question 29b). The final four survey questions had response rates of 60–73%. Table 2 shows the percentage of participants who answered each question. All review questions received an agreement percentage of ≥86% (*n* = 12) for the positively phrased statements, and 14% (*n* = 2) agreed with the statement “The survey took too long” (Figure 1). All participants agreed that the survey was easy to understand and that the survey was important. One participant answered the survey as not easy to complete, and one found the survey not interesting. Of the twelve participant free text comments, eight were positive and four commented that they disliked the question on diets tried and how well they worked because it contained a forced choice where they had to select answers, even if a particular type of diet was not applicable to them. Subsequently, the answer mode for this question was changed. No other changes were made following the pilot survey test.

## 4. Discussion

This study developed and tested the content validity of a questionnaire. Content validity assesses whether the content of a test or instrument is measuring the constructs of interest (barriers to weight loss, perception of diabetes risk, and views of diet strategies following GDM) [18]. Content validity was achieved in this study through acceptable levels of expert agreement in the areas of relevance, clarity, coherence and item response options, and a good response rate with positive feedback from the pilot test stage. The overall CVI value from the expert review also shows good relevance to the constructs being measured. Together, results from the expert review suggest that the elements in the survey are appropriate to assess the constructs of interest.

Items’ clarity and response options received the greatest number of I-CVI scores of ≤0.78 (10 and 12, respectively), while items’ relevance and coherence only received an I-CVI score of ≤0.78 for one and three items, respectively. This suggests that initial issues with the survey at the expert review stage were not related to the question itself but more how the question and response options were worded. The pilot test showed promising results, with a high response rate for most of the questions and high agreement ratios to the survey review questions, including the survey being easy to understand and easy to complete. Results from the pilot test suggest that the final survey questions were appropriate for the target population.

The final survey tool consists of 30 questions, including 6 questions linked to the TDF, with the purpose to assess women’s barriers to weight loss, perception of diabetes risk and views of diet strategies, and services following gestational diabetes in a cross-sectional study. The consequences of T2DM are substantial and include a risk of developing chronic complications including cardiovascular disease, kidney disease, cancer, and early death [1,19]. With the growing percentage of women being diagnosed with GDM [20] and a nearly ten-fold increased risk of these women developing T2DM [2], understanding the barriers to weight loss, perception of disease risk, and views on different diet strategies is a critical step needed to design appropriate diabetes prevention interventions. The TDF provides a validated way to categorize constructs of behavior change and pinpoint areas to focus intervention on such as providing education to increase knowledge or teaching the target population self-monitoring techniques to improve behavioral regulation [9].

Daily energy restriction, although an effective strategy for weight loss, is difficult to adhere to in the long term [21]. Alternative weight loss strategies such as intermittent fasting have become popular in recent years, appealing to many people due to the increased flexibility for eating on non-fasting days [22]. Research to date suggests that an intermittent diet is a suitable alternative to continuous energy restriction for overweight adults, producing comparable weight loss and metabolic improvements to a conventional daily diet [23,24,25]; however, more research in the area is needed, particularly focusing on longer-term outcomes. This survey adds to the body of research on intermittent fasting by investigating the popularity and opinions of this diet strategy in a population of women who are at high risk for developing T2DM.

The main limitation in the development of this survey was that no second round of expert evaluation was undertaken; therefore, experts did not have the opportunity to respond to the changes made following their initial review. However, results from this stage showed acceptable I-CVI scores for the survey items as well as a good overall CVI. Most of the changes made to the survey following the expert review were minor, involving modification of question wording for clarity and coherence as well as changing the response options or mode and changing the survey platform to a more user-friendly interface and allowing the skip logic function. The survey assessed socioeconomic diversity by gathering postcode data; however, questions regarding ethnicity were not included in the survey. This would be worthwhile data to collect in future studies given the large Asian population in Australia, the increased risk of GDM in these women, and a lower BMI threshold where the risk of comorbidities is increased [26]. One of the main goals for GDM management is to control weight gain during pregnancy. The focus of this survey is on weight loss following a GDM pregnancy rather than GDM management; therefore, we did not include questions regarding weight gain in pregnancy or pre-pregnancy weight status. We did assess current BMI and asked whether participants had lost the weight they gained in pregnancy. However, pre-pregnancy weight and weight gain in pregnancy could influence the perception of diabetes risk and would be worth including these questions in future studies.

The final survey will be administered as an online cross-sectional survey to women with previous GDM. The TDF will help to identify barriers to weight loss, perception of diabetes risk and diet strategies that are found to be achievable based on different family circumstances and years past GDM.

## Figures and Tables

**Table 1:** Content validation index (I-CVI) scores from expert evaluation for each item and changes made *.

Version 1 of Survey: Items Constructed by the Research Team						Version 2 of Survey: Items MODIFIED Following Expert Review (Administered to Pilot Test)	
Question No.	Question	Relevance	Clarity	Coherence	Response Options	New Question No.	Changes Made Following Expert Review
1	Informed consent	N/A	N/A	N/A	N/A	1	
2	Have you ever been diagnosed with gestational diabetes during a pregnancy?	1.00	1.00	1.00	1.00	2	Definition of gestational diabetes added. Responses changed to agree to all inclusion criteria.
3	Did you have either type 1 or type 2 diabetes before your gestational diabetes pregnancy?	**0.67**	0.83	**0.67**	**0.67**	2	Question wording changed and merged into the eligibility question (Question 2)
4	Are you aged 18 years or older?	1.00	1.00	1.00	1.00	2	merged into one eligibility question (Question 2)
5	Do you live in Australia?	1.00	1.00	1.00	1.00	2	merged into one eligibility question (Question 2)
6	What is your postcode?	1.00	1.00	1.00	1.00	3	Wording of why postcode data are collected slightly modified
7	Are you currently pregnant?	1.00	1.00	1.00	1.00	6	
8	In what year were you diagnosed with gestational diabetes? (If you have had gestational diabetes more than once, enter your most recent diagnosis)	0.83	1.00	1.00	1.00	15	
9	Did you require insulin injections during your gestational diabetes pregnancy to manage the diabetes?	1.00	**0.67**	1.00	**0.67**	16	Wording and responses changed
10	Since your gestational diabetes pregnancy, have you been diagnosed or told by a doctor or other healthcare professional that you have type1 diabetes, type 2 diabetes, prediabetes, or impaired glucose tolerance?	0.83	1.00	1.00	1.00	17	Wording modified slightly
11	Do you have a family history of diabetes?	0.83	1.00	1.00	1.00	14	Dropdown answer changed to radio buttons
12	When was your youngest child born? (in what month and year did your most recent pregnancy end)	0.83	**0.50**	0.83	**0.67**	8	Wording modified
13	How many children do you have living at home with you?	1.00	**0.67**	1.00	0.83	7	Wording modified
14	What is your age in years?	1.00	1.00	1.00	1.00	5	
15	What is your height in cm without shoes on? (If you do not know your height in centimeters (cm), you can use the following link to convert your height from feet and inches into cm)	1.00	1.00	1.00	**0.67**	9	Included hyperlink to convert height from imperial to metric units
16	What is your current weight in kilograms (kg) without shoes and in light or no clothing? (If you have a set of bathroom scales, please weigh yourself and enter your weight in here. If you are unable to weight yourself but you know how much you weigh, please use that weight)	1.00	0.83	1.00	1.00	10	Wording modified and hyperlink added
17	For the last question about your weight, did you weigh yourself or estimate your weight?	1.00	1.00	1.00	1.00	11	
18	What is your employment status?	1.00	1.00	1.00	0.83	4	Added ‘other’ option
19	Do you think you are at risk of developing type 2 diabetes?	1.00	**0.67**	0.83	**0.67**	13	Answer mode and options changed
20	Do you consider yourself to be under, within, or over a healthy weight?	0.83	0.83	1.00	**0.67**	12	Answer options changed
21	Since you have had gestational diabetes, have you been told by a doctor or other healthcare professional (e.g., a dietitian, midwife, diabetes nurse, etc.) that you need to lose weight?	0.83	**0.67**	1.00	**0.67**	18	Wording modified
22	Have you lost, gained, or maintained your weight since your last baby was born?	0.83	**0.50**	1.00	0.83	22	Wording modified
23	Since your gestational diabetes pregnancy, have you gone on any diets or tried to lose weight?	1.00	1.00	1.00	1.00	23	Wording slightly modified based on TDF fit
24	Have you tried any of the following diets, programs, or procedures since having gestational diabetes?	1.00	**0.50**	**0.67**	0.83	24	Merged Q24 and Q25 and wording changed
25	Are there any other types of diets or weight loss programs or services you have tried for weight loss? (please specify what you have tried and if it worked for you or not)	1.00	1.00	1.00	0.83	24	Merged Q24 and Q25 and wording changed
26	How long after having a baby do you think it is safe to start to try and lose weight?	1.00	0.83	1.00	**0.60**	20	Answer mode and responses changed
27	Do you think it is safe to lose weight while you are breastfeeding?	1.00	1.00	1.00	0.83	19	Added ‘I don’t know’ option
28	Did you breastfeed your baby after your gestational diabetes pregnancy?	1.00	0.83	1.00	**0.67**	21	Answer options changed
29	Please read the following statements about weight loss and answer each question how much you agree or disagree with the statement	1.00	**0.67**	1.00	**0.5**	25	Merged Q29 and 30. Answer wording ‘neither agree or disagree’ changed to ‘not sure’ for simplicity
30	Are there any other things not listed above that might make weight loss hard for you right now?	1.00	1.00	1.00	1.00	25	Merged Q29 and 30.
31	The following statements will ask you what kind of weight loss program or service you think might work for you at the moment. Please read each statement and answer according to how much you agree or disagree that each one could work for you.	1.00	0.83	1.00	0.83	26	Wording slightly changed and question merged with Q32
32	Are there any other things you think could help you to lose weight right now?	1.00	0.83	0.83	1.00	26	Merged with Q31
33	Where would you go for information on how to lose weight?	1.00	1.00	1.00	0.83	27	Wording changed “Where have you or would you go to look for information on how to lose weight?”
34	Have you heard of the 5:2 diet?	1.00	**0.67**	1.00	0.83	28	wording changed (This is also known as an intermittent or fasting diet)
35	If you answered ‘yes’ to the last question, have you ever tried the 5:2 diet?	1.00	0.83	1.00	**0.67**	29	typographical error corrected. Skip logic added to the previous question
36	Do you think the 5:2 diet could be a good option for you right now? Please feel free to comment if you would like to add any further thoughts.	0.83	**0.67**	**0.50**	**0.67**	30	Wording changed

* Items that received a score of ≤0.78 are shown in boldface.

**Table 2:** Number and percentage of questions answered for each item in the pilot test.

Question No.	Question	No. Presented with Question	No. Who Answered Question	% Answered
1	Consent	21	21	100%
2	Eligibility	21	21	100%
3	Postcode	20	18	90%
4	Employment status	20	20	100%
5	Age	20	20	100%
6	Pregnant	20	20	100%
7	Number of children at home	20	20	100%
8	Year youngest child born	20	20	100%
9	Height	20	20	100%
10	Weight	20	18	90%
11	Weight measured or estimated	20	20	100%
12	Perception of weight status	20	20	100%
13	Perception of diabetes risk	20	19	95%
14	Family history of diabetes	20	19	95%
15	Year diagnosed with GDM	20	19	95%
16	Medication during GDM pregnancy	20	19	95%
17	Diagnosis of diabetes since GDM	20	19	95%
18	Has a healthcare professional told you to lose weight?	20	19	95%
19	Do you think it is safe to lose weight while breastfeeding?	20	19	95%
20	How long after having a baby do you think it is safe to start trying to lose weight (breastfeeding and not breastfeeding)?	20	19	95%
21	Did you breastfeed your baby after your GDM pregnancy?	20	19	95%
22	Has your weight changed since your last baby was born?	20	19	95%
23 *	Since having GDM, have you thought about or tried losing weight?	20	19	95%
24	Diets tried and how well they worked?	13	13	100%
25	Barriers to weight loss (Likert scale)	20	18	90%
26	Weight loss programs or services tried	20	18	90%
27	Where would you go for information on how to lose weight?	20	18	90%
28 *	Have you heard of the 5:2 diet?	20	18	90%
29a	Have you tried the 5:2 diet?	15	11	73%
29b	Do you think the 5:2 diet could be a good option for you?	5	3	60%
30	Pilot review question (ease to complete, understand, length and importance) (Likert scale)	20	14	70%
31	Pilot review question—We would like to hear your thoughts about the survey	20	12	60%

* Skip logic used at this question. GDM: gestational diabetes.

## Data Availability

The data presented in this study are available on request from the corresponding author.

## Appendix A

**Table A1:** The Theoretical Domains Framework domains and constructs *.

Domain	Constructs
1. Knowledge	Knowledge, Procedural knowledge, Knowledge of task environment
2. Skills	Skills, Skills Development, Competence, Ability, Interpersonal skills, Practice, Skills assessment
3. Social/Professional Role and identity	Professional identity, Professional role, Social identity, Identity, Professional boundaries, Professional confidence, Group identity, Leadership, Organizational commitment
4. Beliefs about Capabilities	Self-confidence, Perceived competence, Self-efficacy, Perceived behavioral control, Beliefs, Self-esteem, Empowerment, Professional confidence
5. Optimism	Optimism, Pessimism, Unrealistic optimism, Identity
6. Beliefs about Consequences	Beliefs, Outcome expectancies, Characteristics of outcome expectancies, Anticipated regret, Consequents
7. Reinforcement	Rewards, Incentives, Punishment, Consequents, Reinforcement, Contingencies, Sanctions
8. Intentions	Stability of intentions, Stages of change model, Transtheoretical model and stages of change
9. Goals	Goals (distal/proximal), Goal priority, Goal/target setting, Goals (autonomous/controlled), Action planning, Implementation intention
10. Memory, attention, and decision processes	Memory, Attention, Attention control, Decision making, Cognitive overload/tiredness
11. Environmental context and resources	Environmental stressors, Resources/material resources, Organizational culture/climate, Salient events/critical, incidents, Person × environment interaction, Barriers and facilitators
12. Social Influences	Social pressure, Social norms, Group conformity, Social comparisons, Group norms, Social support, Power, Intergroup conflict, Alienation, Group identity, Modeling
13. Emotion	Fear, Anxiety, Affect, Stress, Depression, Positive/negative affect, Burn-out
14. Behavioral Regulation	Self-monitoring, Breaking habit, Action planning

* Adapted from Cane J, O’Connor D and Michie S, 2012 (1).
